# CSANet: Context–Spatial Awareness Network for RGB-T Urban Scene Understanding

**DOI:** 10.3390/jimaging11060188

**Published:** 2025-06-09

**Authors:** Ruixiang Li, Zhen Wang, Jianxin Guo, Chuanlei Zhang

**Affiliations:** 1 School of Electronic Information, Xijing University, Xijing Road, Chang’an District, Xi’an 710123, China; 20150157@xijing.edu.cn (R.L.); guojianxin@xijing.edu.cn (J.G.); 2 School of Computer Science, Northwestern Polytechnical University, Dongxiang Road, Chang’an District, Xi’an 710129, China; 3 School of Artificial Intelligence, Tianjin University of Science and Technology, Dagu South Road, Hexi District, Tianjin 300457, China; 97313114@tust.edu.cn

**Keywords:** RGB-T semantic segmentation, urban scene understanding, multi-modal fusion, encoder–decoder structure, attention mechanism

## Abstract

Semantic segmentation plays a critical role in understanding complex urban environments, particularly for autonomous driving applications. However, existing approaches face significant challenges under low-light and adverse weather conditions. To address these limitations, we propose CSANet (Context Spatial Awareness Network), a novel framework that effectively integrates RGB and thermal infrared (TIR) modalities. CSANet employs an efficient encoder to extract complementary local and global features, while a hierarchical fusion strategy is adopted to selectively integrate visual and semantic information. Notably, the Channel–Spatial Cross-Fusion Module (CSCFM) enhances local details by fusing multi-modal features, and the Multi-Head Fusion Module (MHFM) captures global dependencies and calibrates multi-modal information. Furthermore, the Spatial Coordinate Attention Mechanism (SCAM) improves object localization accuracy in complex urban scenes. Evaluations on benchmark datasets (MFNet and PST900) demonstrate that CSANet achieves state-of-the-art performance, significantly advancing RGB-T semantic segmentation.

## 1. Introduction

Semantic segmentation, a vital branch of computer vision (CV), aims to classify each pixel in an image into a specific category [1,2]. It has been widely applied in fields such as autonomous driving [3], urban planning [4], and intelligent transportation [5]. However, existing optical image-based semantic segmentation methods face significant challenges in autonomous driving scenarios: (1) segmentation performance often degrades in complex street scenes, particularly for small objects [6,7,8]; and (2) segmentation accuracy is severely impacted under adverse conditions such as low light, fog, rain, and snow [9,10,11]. Advancements in sensor technology have improved access to infrared waveband information, making thermal imaging a robust solution for perception and understanding in driving environments. Thermal infrared (TIR) imaging provides unique advantages for semantic segmentation in urban environments, such as robustness to variable lighting conditions and the ability to detect heat-emitting objects regardless of ambient illumination. However, TIR sensors also have intrinsic limitations. Their sensitivity to surface emissivity can cause material-dependent variations in thermal signatures, while environmental temperature changes and sensor-inherent thermal noise can further affect image quality and reliability. Moreover, TIR images often lack fine texture details present in RGB images, potentially reducing segmentation accuracy for small or visually similar objects. These factors highlight the necessity for carefully designed fusion strategies to fully leverage the complementary properties of RGB and TIR modalities in challenging real-world scenarios [12,13,14]. The primary challenge in multi-modal image processing lies in the effective extraction and fusion of features from different modalities.

**Figure 1:**
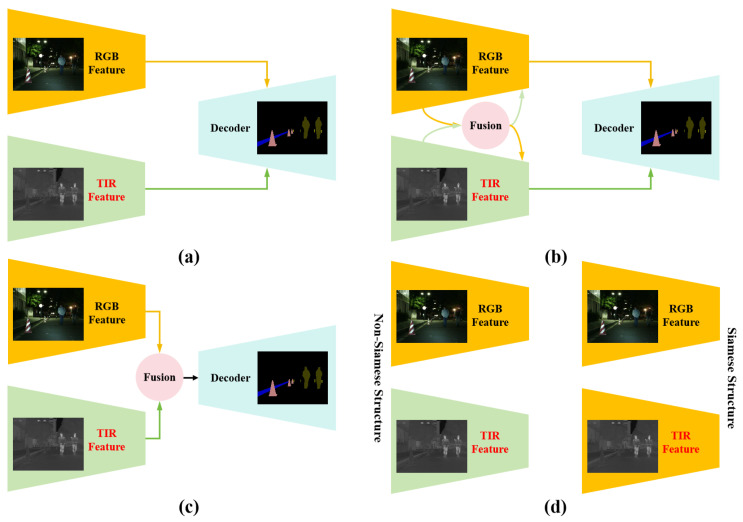
Different multi-modal feature extraction and fusion strategies. (**a**) Direct feature fusion in the decoder module; (**b**) Parallel feature extraction and fusion during processing; (**c**) Separate stages for feature extraction, fusion, and decoding; (**d**) Comparison between non-Siamese and Siamese structures for multi-modal feature extraction.

Currently, multi-modal feature fusion paradigms can be categorized into three types, as illustrated in Figure 1: (a) direct feature fusion in the decoder module [15,16]; (b) parallel feature extraction and fusion during processing [17,18,19]; and (c) separate stages for feature extraction, fusion, and decoding [20,21]. While these paradigms are widely applied in RGB-T semantic segmentation, the first paradigm struggles to ensure feature complementarity, and the second risks feature confusion. To address these limitations, we adopt the third paradigm, which carefully integrates the unique characteristics of multi-modal features. This approach not only facilitates effective feature complementarity in subsequent fusion stages but also exploits the synergy of features at different levels, thereby improving overall model performance. Furthermore, as shown in Figure 1d, multi-modal feature extraction strategies can be classified into two categories: Siamese structures with shared parameters [22,23] and non-Siamese structures without shared parameters [16,24]. Given that RGB images provide color information of natural scenes while TIR images capture infrared waveband data, shared parameter structures often fail to effectively exploit their complementary properties. Existing studies demonstrate that convolutional neural networks (CNNs) excel at extracting local fine-grained features [25], whereas Transformer models leverage self-attention mechanisms to capture long-range dependencies [26]. Based on these observations, we propose a non-Siamese hybrid structure combining CNNs and Transformers to extract and integrate multi-modal features from RGB-T data effectively.

**Figure 2:**
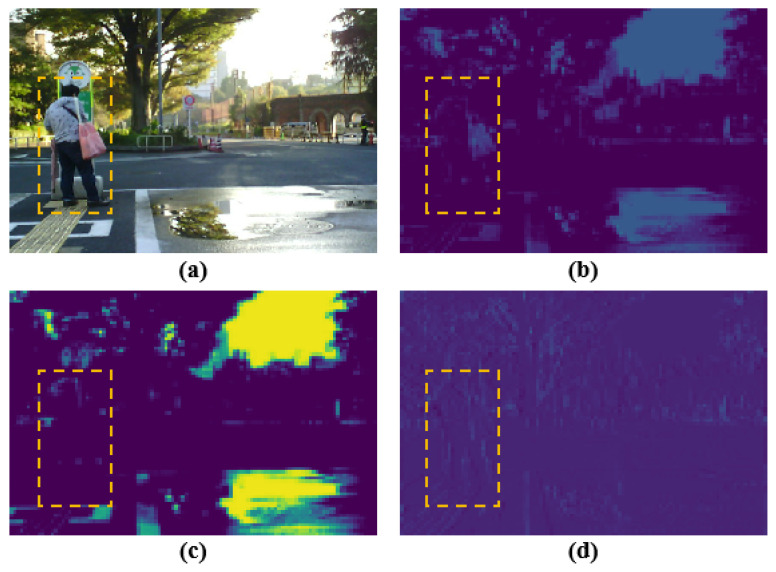
Feature visualization of different feature fusion methods, including (**a**) original images, (**b**) feature redundancy, (**c**) feature loss, (**d**) effective feature.

To fuse feature maps of different modalities and scales, existing feature fusion strategies include element-wise addition, element-wise multiplication, feature concatenation, and attention mechanisms. Element-wise addition [27] directly merges features, which enhances model robustness, while element-wise multiplication [28] emphasizes feature selection and shared information. Feature concatenation [29] preserves the integrity of original features, enabling the model to effectively understand the semantic information of multi-modal data. However, as shown in Figure 2, these three fusion methods are prone to feature redundancy or information loss. As a novel feature fusion approach, the attention mechanism [30] extracts important regional features and achieves fine-grained fusion. Therefore, in this study, we design attention mechanisms to achieve efficient fusion of multi-modal features. Additionally, feature extraction in the model can be categorized into shallow local features and deep semantic features. As the network depth increases, the size of feature maps gradually decreases [31]. To enhance model performance, shallow and deep features must be selectively fused to mitigate redundant feature interference. Based on these insights, CSANet incorporates the Channel-Space Cross-Fusion Module (CSCFM) and the Multi-Head Fusion Module (MHFM) to address the enhancement and fusion of shallow local features and deep semantic information. Meanwhile, the Spatial Coordinate Attention Mechanism (SCAM) is introduced to improve the localization precision of moving objects in complex urban scenes. The contributions of this study are summarized as follows:

- By fully exploring the feature differences between multi-modal data, we propose a novel Context Spatial Awareness Network (CSANet) for RGB-T semantic segmentation, achieving state-of-the-art performance on the MFNet and PST900 datasets.
- We design the CSCFM to extract shallow fine-grained local features from multi-modal data and the MHFM to capture and enhance deep semantic feature information. A cross-stage, multi-scale hierarchical framework is employed to efficiently fuse features across modalities.
- The SCAM is introduced into the encoder structure of CSANet to improve the localization accuracy of moving objects in complex scenes. Additionally, a lightweight decoder framework is used to restore multi-modal feature resolution while reducing computational complexity.

The remainder of this article is organized as follows: Section 2 reviews the related work. Section 3 presents the proposed CSANet framework. Section 4 discusses experimental analyses, and Section 5 concludes the study.

## 2. Related Works

### 2.1. RGB Semantic Segmentation

With the development of convolutional neural networks (CNNs), Long et al. [32] first introduced fully convolutional networks (FCNs) for semantic segmentation, proposing an end-to-end, pixel-to-pixel training approach. To enhance the extraction of image details and edge information, Noh et al. [1] proposed a deconvolution network for semantic segmentation tasks. However, early semantic segmentation methods faced challenges such as high memory consumption, low computational efficiency, and limited accuracy. To address these issues, Badrinarayanan et al. [33] introduced SegNet, an encoder–decoder architecture designed to progressively restore pixel details. Early models often relied on relatively shallow networks, such as VGGNet [31], but deeper networks introduced issues like gradient vanishing or explosion. To overcome this, He et al. [26] proposed ResNet, utilizing residual learning to enable deeper networks and improve learning capacity. For complex scene segmentation, the DeepLab series [34,35,36,37] employed dilated convolutions with varying rates to expand the receptive field, enabling the capture of multi-scale visual features. The UNet series [38,39,40], on the other hand, utilized contraction and symmetrical extension paths to integrate contextual information. For example, UNet++ [40] improved upon UNet by introducing nested and dense skip connections, further enhancing segmentation performance. The Transformer model [41] revolutionized natural language processing tasks, and Dosovitskiy et al. [42] extended its application to computer vision, demonstrating its versatility. Strudel et al. [43] applied Transformers to semantic segmentation by integrating the Vision Transformer (ViT) as the core component of a segmentation pipeline. ViT [34] processes input images as patch sequences, capturing global dependencies more effectively than traditional CNN-based convolutions, making it advantageous for global feature extraction and enhancement. Despite its strengths, ViT’s effectiveness relies heavily on access to large-scale training datasets, limiting its ability to extract local features as efficiently as CNNs. For RGB-T semantic segmentation, which often involves smaller datasets and requires detailed local visual information in low-illumination conditions, constructing a ViT model suitable for RGB-T remains a challenge. Additionally, the computational resource requirements of ViT pose constraints for deployment in scenarios with limited hardware capabilities. To address these challenges, hybrid models integrating CNNs and Transformers have been proposed. By combining CNNs’ strength in extracting local features with Transformers’ ability to capture global dependencies, these models achieve a balance between computational efficiency and segmentation performance. Furthermore, the introduction of the multi-head attention mechanism significantly reduces the computational complexity of ViT, making it more scalable for resource-limited scenarios while maintaining high performance.

### 2.2. RGB-T Semantic Segmentation

Thermal infrared (TIR) images capture the thermal information of scene objects, which is perceived by the human brain as spatial information. In depth maps, however, different segmentation regions may share the same depth values without distinct features. Ha et al. [44] first introduced MFNet, which employs a dual-stream architecture to extract RGB-T features, followed by cascaded fusion to accomplish semantic segmentation tasks. RTFNet [17] and FuseSeg [45] adopt architectures similar to MFNet but rely on element-wise addition for feature fusion. Shivakumar et al. [46] proposed a cross-level fusion structure that integrates feature maps from different levels of RGB and TIR images. However, these methods predominantly use simple fusion strategies, such as element-wise addition and concatenation, to capture cross-modal features. Such approaches often result in information redundancy by neglecting the inherent differences between RGB and TIR modalities. More sophisticated RGB-T semantic segmentation methods, such as those in [12,16], emphasize structural classification of features across different stages of the model. By designing hierarchical fusion strategies and integrating multi-level supervision, these methods achieve a refined understanding of spatial and structural information. These approaches not only enhance recognition accuracy for object boundaries and positions but also highlight the significance of layer-specific fusion strategies and multi-task supervision in RGB-T semantic segmentation. Recently, Zhang et al. [14,20,47] combined CNNs with Transformers for feature extraction and fusion, significantly improving multi-modal semantic segmentation accuracy. The research community has increasingly adopted models like Swin Transformer [48] and SegFormer [49] as encoders or for feature fusion, further enhancing RGB-T semantic segmentation performance. These methods integrate traditional CNN-based techniques with Transformer-based attention mechanisms, providing more powerful and nuanced feature representations for complex segmentation tasks. The Swin Transformer [48], with its hierarchical Transformer structure, efficiently processes multi-scale features. By employing a sliding window mechanism, it reduces the computational complexity of self-attention, enabling the model to handle images of varying sizes. SegFormer [49], on the other hand, combines the self-attention mechanism with multi-scale feature extraction. Its lightweight design and strong performance enable it to handle high-resolution inputs while maintaining a low computational cost. These developments represent a significant step forward in RGB-T semantic segmentation, balancing computational efficiency with accuracy.

## 3. Methodology

**Figure 3:**
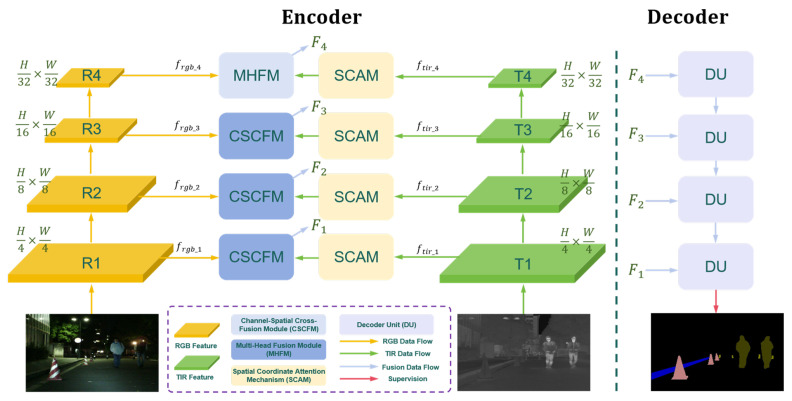
The architecture of our CSANet. The left side comprises the Encoder module, while the right side encompasses thedecoder module. The encoder module integrates different feature extraction and fusion modules to enhance visual and positional information. The decoder module employs multiple supervisions to improve the accuracy of model prediction. Upon feeding multi-modal images into the encoder to extract hierarchical features T/R1, T/R2, T/R3, T/R4, the encoder includes the modules CSCFM, MHFM, and SCAM, designed to amalgamate these hierarchical features into F1, F2, F3, and F4, respectively. Subsequently, the fused features $F_{i}$ are inputted into the decoder for progressive pixel restoration. The framework aims to enhance the effectiveness of the encoder–decoder structure in RGB-T semantic segmentation tasks by using multi-modal feature complementarity.

To efficiently achieve RGB-T semantic segmentation in urban scenes, we propose a Context Spatial Awareness Network (CSANet), as illustrated in Figure 3. CSANet is designed to effectively learn local and global features from RGB and TIR images while performing fine-grained fusion of multi-modal features. Specifically, CSANet incorporates three key modules: the Channel–Spatial Cross-Fusion Module (CSCFM), which enhances the representation of visual information by fusing multi-modal features; the Multi-Head Fusion Module (MHFM), which performs global feature modeling and multi-modal feature calibration; and the Spatial Coordinate Attention Mechanism (SCAM), which improves the representation of positional information within object regions. Additionally, the final-stage feature fusion promotes interaction between visual and positional information, enhancing the model’s generalization capability.

For the encoder of CSANet, we adopt PVTv2 [50,51] as the backbone network, referred to as PVE. RGB and TIR image pairs (denoted as $P_{\mathrm{RGB}}$ and $P_{\mathrm{TIR}}$, respectively) from the same scene are input into the encoder network to extract multi-modal features across four stages without parameter sharing. In the first stage, the feature map size is scaled to 1/4 of the original image size. In the subsequent three stages, the feature map size is halved successively. This hierarchical scaling of feature sizes across stages progressively refines features, enabling the model to focus on abstract and semantic representations as the network depth increases. This strategy balances computational efficiency and enhances the extraction of multi-scale feature information. The extracted RGB and TIR features at each stage are represented as follows:

(1) $$\begin{array}{c} f_{\mathrm{rgb}\_i}=\mathrm{PVE}\left(P_{\mathrm{RGB}}\right),\;i=1,2,3,4\\ f_{\mathrm{tir}\_i}=\mathrm{PVE}\left(P_{\mathrm{TIR}}\right),\;i=1,2,3,4 \end{array}$$

where $f_{\mathrm{rgb}\_i}$ and $f_{\mathrm{tir}\_i}$ represent the RGB and TIR features extracted at the *i*-th stage of the encoder, respectively. The PVE backbone combines the strengths of CNNs for local feature extraction and Transformers for capturing global context dependencies, as described in [51]. By integrating linear-time attention mechanisms, PVE significantly enhances model performance while balancing computational efficiency. To obtain enhanced fine-grained visual features $C_{i}$, the RGB and TIR features from the same stage are input into the CSCFM. Spatial attention mechanisms are applied to enrich the TIR features with visual information, effectively addressing segmentation challenges for multi-scale objects in low-illumination conditions. The overall feature extraction process is as follows:

(2) $$\begin{array}{c} C_{i}=F_{\mathrm{CSCFM}}\left(f_{\mathrm{rgb}\_i},f_{\mathrm{tir}\_i}\right),\;i=1,2,3\\ M_{i}=F_{\mathrm{MHFM}}\left(f_{\mathrm{rgb}\_i},f_{\mathrm{tir}\_i}\right),\;i=4\\ S_{i}=F_{\mathrm{SCAM}}\left(f_{\mathrm{tir}\_i}\right),\;i=1,2,3,4 \end{array}$$

In the final stage of feature extraction, MHFM is used to fuse RGB and TIR features, enabling refined global feature modeling. Additionally, SCAM is integrated into different stages of the feature extraction process to improve localization accuracy for objects of varying scales, leveraging TIR-based positional information. This multi-stage integration ensures accurate and robust segmentation performance in complex urban scenes. The process of the proposed method is shown in Algorithm 1.
**Algorithm 1** CSANet: RGB-T Semantic Segmentation**Require:** RGB image $P_{\mathrm{RGB}}\in R^{3\times H\times W}$, TIR image $P_{\mathrm{TIR}}\in R^{3\times H\times W}$.**Ensure:** Segmentation map $M\in R^{n\_cls\times H\times W}$.
  1:Extract multi-stage features for RGB and TIR:  2:**for** each stage i∈{1,2,3,4} **do**  3:   Extract RGB features: $f_{\mathrm{rgb}\_i}=\mathrm{PVE}\left(P_{\mathrm{RGB}},i\right)$.  4:   Extract TIR features: $f_{\mathrm{tir}\_i}=\mathrm{PVE}\left(P_{\mathrm{TIR}},i\right)$.  5:**end for**  6:**for** each stage i∈{1,2,3} **do**  7:   Fuse features using CSCFM: $C_{i}=F_{\mathrm{CSCFM}}\left(f_{\mathrm{rgb}\_i},f_{\mathrm{tir}\_i}\right)$.  8:   Enhance positional information using SCAM: $S_{i}=F_{\mathrm{SCAM}}\left(f_{\mathrm{tir}\_i}\right)$.  9:**end for**10:Fuse features at final stage using MHFM:11:$M_{4}=F_{\mathrm{MHFM}}\left(f_{\mathrm{rgb}\_4},f_{\mathrm{tir}\_4}\right)$.12:Enhance positional information at final stage:13:$S_{4}=F_{\mathrm{SCAM}}\left(f_{\mathrm{tir}\_4}\right)$.14:Combine multi-stage features:15:$F_{\mathrm{fuse}}={\left\{C_{i},S_{i}\right\}}_{i=1}^{3}\cup \left\{M_{4},S_{4}\right\}$.16:Decode fused features:17:$M=\mathrm{Decoder}(F_{\mathrm{fuse}})$.18:**return** Segmentation map M.

### 3.1. Channel–Spatial Cross-Fusion Module

**Figure 4:**
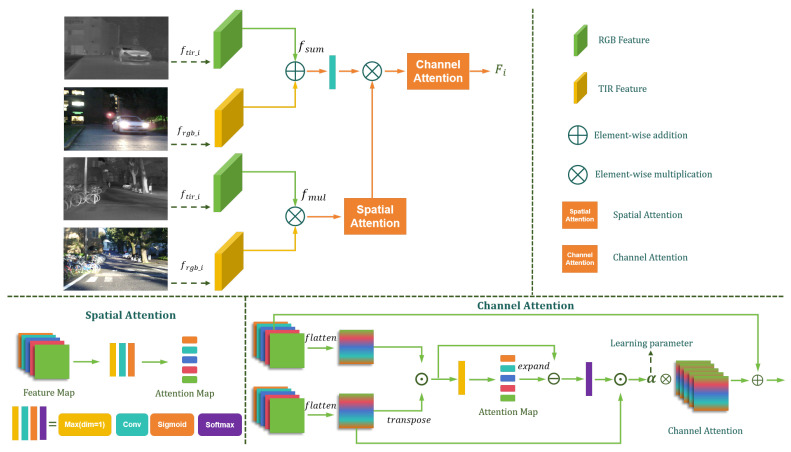
The detailed architecture of the Channel–Spatial Cross-Fusion Module (CSCFM).

The visual features play a crucial role in pixel restoration and model cognition, as the specific category of a pixel is predominantly determined by the visual information. Therefore, the fusion and enhancement of shallow visual features are critical for improving model prediction performance. To fully capture the visual features of RGB-T data, we propose the Channel–Spatial Cross-Fusion Module (CSCFM), whose specific structure is shown in Figure 4.

Specifically, we first perform addition and multiplication operations on the input features $f_{\mathrm{rgb}\_i}$ and $f_{\mathrm{tir}\_i}$ to obtain $f_{\mathrm{sum}\_i}$ and $f_{\mathrm{mul}\_i}$, respectively. The $f_{\mathrm{mul}\_i}$ feature highlights shared salient features and compensates for the deficiencies in each modality. Next, $f_{\mathrm{mul}\_i}$ is processed using the spatial attention mechanism to extract local details and enhance the connections between different regions within the image. Subsequently, the convolution-processed $f_{\mathrm{sum}\_i}$ and $f_{\mathrm{mul}\_i}$ are multiplied and input into the channel attention mechanism to produce the fused feature $F_{i}$. This process adjusts the contribution of feature channels, enhancing the generalization ability and robustness of the model.

(3) $$F_{i}=\mathrm{CA}\left(\mathrm{Conv}\left(f_{\mathrm{sum}\_i}\right)\times \mathrm{SA}\left(f_{\mathrm{mul}\_i}\right)\right),\;i=1,2,3$$

where CA(·) and SA(·) represent the channel attention and spatial attention functions, respectively. The spatial attention mechanism aims to enhance visual detail features by emphasizing the most significant values in each feature map. Specifically, CSCFM extracts the maximum value of each feature map along the one-dimensional channel to construct the attention map and employs an activation function to prevent gradient explosion, ensuring stable and efficient feature enhancement. The calculation is given by

(4) $$\mathrm{SA}=\sigma \left(\mathrm{Conv}\left(\mathrm{Max}\left(f_{\mathrm{mul}\_i}\right)\right)\right)$$

where Conv(·) and Max(·) denote convolution and maximum pooling operations, respectively. The channel attention mechanism distinguishes the saliency between feature maps to enhance feature extraction and channel modeling capabilities. First, the input feature $f\in R^{B\times C_{i}\times H_{i}\times W_{i}}$ is reshaped to $\tilde{f}\in R^{B\times C_{i}\times d}$, where $d=H_{i}\times W_{i}$. The matrix product of $\tilde{f}$ and its transpose is computed to obtain the relation matrix $\tilde{w}$:

(5) $$\tilde{w}=\tilde{f}⊙\mathrm{Transpose}\left(\tilde{f}\right),\;\tilde{w}\in R^{B\times C_{i}\times C_{i}}$$

where $\tilde{w}$ represents the relation matrix. Next, the maximum relationship value $\tilde{v}$ of each channel in $\tilde{w}$ relative to other channels is computed, corresponding to the autocorrelation coefficient of the channel. The maximum relationship value is extended to match the dimension of $\tilde{w}$, and an element-wise subtraction is performed to obtain the reverse maximum relationship coefficient matrix *E*. The enhanced attention-weighted feature ${\tilde{f}}_{w}$ is then computed using the softmax function and matrix multiplication:

(6) $$\begin{array}{c} \tilde{v}=\mathrm{Max}\left(\tilde{w}\right);\;E=\tilde{v}-\tilde{w}\\ {\tilde{f}}_{w}=E⊙\tilde{f};\;F_{i}=\alpha {\tilde{f}}_{w}+f \end{array}$$

where ${\tilde{f}}_{w}\in R^{B\times C_{i}\times d}$ is restored to the original dimension of $f\in R^{B\times C_{i}\times H_{i}\times W_{i}}$, and α is a learnable parameter for the residual connection. The resulting fused feature $F_{i}$ represents the cross-fused output of CSCFM, integrating salient spatial and channel relationships.

### 3.2. Multi-Head Fusion Module

**Figure 5:**
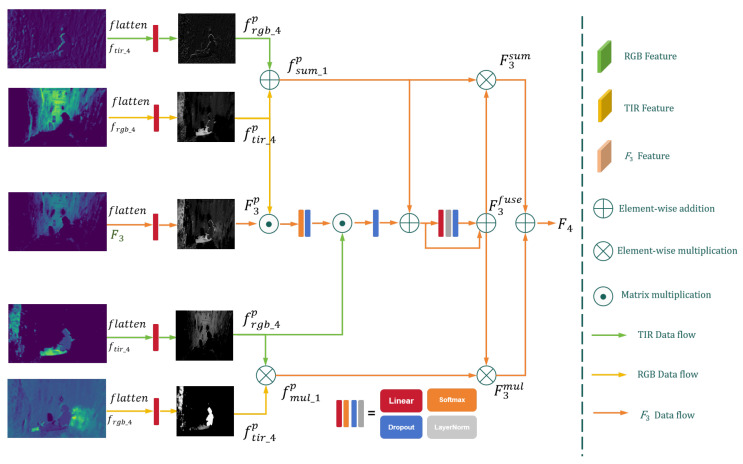
The detailed architecture of Multi-Head Fusion Module (MHFM).

The thermal features of object regions in TIR images can be perceived as positional information, while the semantic features in RGB images not only represent positional information but also play a critical role in pixel-level feature reconstruction. To fully extract the semantic features of multi-modal data, we construct the Multi-Head Fusion Module (MHFM), which fuses multi-modal visual features to enhance the model’s global understanding and refine semantic segmentation results. The structure of MHFM is shown in Figure 5.

Specifically, MHFM takes the input features $\left\{f_{\mathrm{rgb}\_4},f_{\mathrm{tir}\_4},F_{3}\right\}\in R^{B\times C\times H\times W}$, where $f_{\mathrm{rgb}\_4}$ and $f_{\mathrm{tir}\_4}$ are the deep features extracted from the fourth stage of RGB and TIR encoders, and $F_{3}$ is the fused feature from the previous stage. The input features are first flattened and linearly transformed into $\left\{f_{\mathrm{rgb}\_4}^{p},f_{\mathrm{tir}\_4}^{p},F_{3}^{p}\right\}\in R^{B\times C\times H\times W}$, similar to the *Q*, *K*, and *V* matrix transformations in Transformer models. The multi-head attention mechanism is then employed to capture and enhance global contextual features.

(7) $$\begin{array}{c} W_{1}=DP\left(\mathrm{Soft}\max\left(\frac{f_{\mathrm{mul}\_1}^{p}⊙F_{3}^{p}}{\sqrt{C_{4}}}\right)\right)⊙f_{\mathrm{mul}\_1}^{p}\\ W_{2}=DP\left(W_{1}\right)+f_{\mathrm{mul}\_1}^{p} \end{array}$$

where DP(·) denotes the dropout function. The resulting matrix $W_{2}$ is processed by a feedforward neural network to obtain the fused feature $F_{3}^{\mathrm{fuse}}$, which combines visual and semantic features and enhances positional information for object regions:

(8) $$F_{3}^{\mathrm{fuse}}=W_{2}+DP\left(LN\left(\mathrm{Linear}(W_{2})\right)\right)$$

To further integrate visual and positional information, $F_{3}^{\mathrm{fuse}}$ is combined with $F_{3}^{\mathrm{sum}}$ and $F_{3}^{\mathrm{mul}}$ through a weighted multiplication operation:

(9) $$F_{4}=F_{3}^{\mathrm{sum}}\times F_{3}^{\mathrm{fuse}}+F_{3}^{\mathrm{mul}}\times F_{3}^{\mathrm{fuse}}$$

where LN(·) represents the layer normalization function. This process ensures that the model can effectively perceive global positional context and spatial correlations, enabling accurate classification of object-region pixels. To restore the segmentation results to the original resolution, $F_{4}\in R^{B\times 2\times H\times W}$ is upsampled 32 times using linear interpolation. Additionally, a binary image supervision mechanism is introduced to supervise and learn the positional and visual features of the same object region, improving the model’s generalization capability.

### 3.3. Spatial Coordinate Attention Mechanism

**Figure 6:**
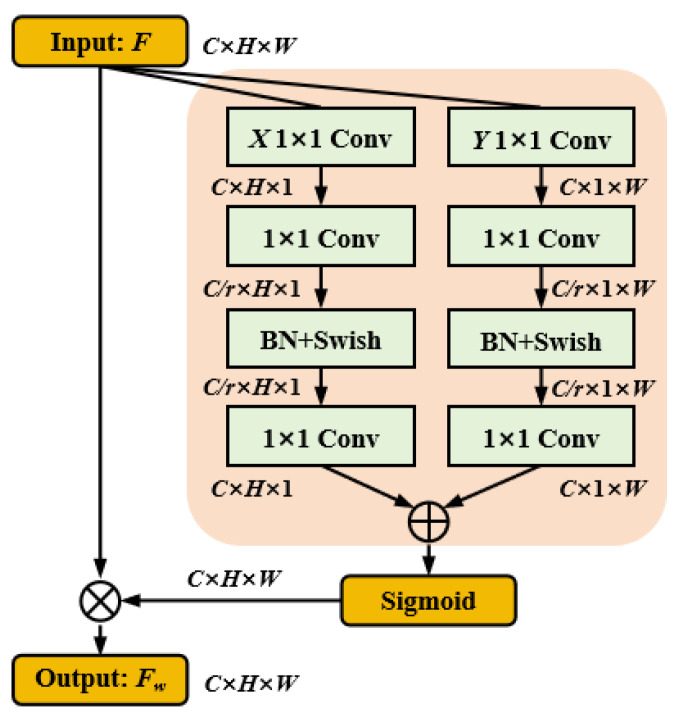
The architecture of the Spatial Coordinate Attention Mechanism.

To fully extract and enhance the positional feature information of RGB-T object regions, inspired by the coordinate attention mechanism [52], we propose the Spatial Coordinate Attention Mechanism (SCAM). SCAM embeds spatial position information into the channel attention feature map by leveraging the interdependence between spatial and channel dimensions, resulting in a three-dimensional attention weight. This attention weight enhances the feature representation of different object categories and extracts local detail information, facilitating accurate object region localization. As shown in Figure 6, let $F\in R^{C\times H\times W}$ denote the input feature map, where *C*, *H*, and *W* represent the number of channels, height, and width, respectively.

To aggregate the feature map *F* along the horizontal direction, we apply a 1×1 convolution to the *X* coordinate, which extracts long-distance dependencies in the horizontal dimension while preserving vertical positional information:

(10) $$z^{x}={\mathrm{Conv}}_{1\times 1}^{X}\left(F\right)$$

where $z^{x}\in R^{C\times H\times 1}$ represents the *X* coordinate feature map. Next, $z^{x}$ is processed through a 1×1 convolution combined with batch normalization and a nonlinear activation function to produce the feature map $f^{X}$:

(11) $$f^{X}=\mathrm{Swish}\left(\mathrm{BN}\left({\mathrm{Conv}}_{1\times 1}\left(z^{x}\right)\right)\right)$$

where Swish(·) is the nonlinear activation function, BN(·) represents batch normalization, and $f^{X}\in R^{C/r\times H\times 1}$ encodes spatial information in the vertical direction, with *r* being the channel compression ratio. The channel dimension of $f^{X}$ is then restored to match the number of channels in *F* using another 1×1 convolution:

(12) $$g^{X}={\mathrm{Conv}}_{1\times 1}\left(f^{X}\right)$$

where $g^{X}$ is the feature map after channel transformation. Similarly, for the *Y* coordinate, 1×1 convolution is applied to aggregate the feature map *F* along the vertical direction, retaining horizontal positional information while capturing long-distance dependencies in the vertical dimension:

(13) $$\begin{array}{c} z^{y}={\mathrm{Conv}}_{1\times 1}^{Y}\left(F\right)\\ f^{Y}=\mathrm{Swish}\left(\mathrm{BN}\left({\mathrm{Conv}}_{1\times 1}\left(z^{y}\right)\right)\right)\\ g^{Y}={\mathrm{Conv}}_{1\times 1}\left(f^{Y}\right) \end{array}$$

After obtaining $g^{X}$ and $g^{Y}$, the broadcast addition operation is performed to combine the two features, followed by the Sigmoid function to compute the spatial coordinate weight β:

(14) $$\beta =\mathrm{Sigmoid}\left(g^{X}⊕g^{Y}\right)$$

where $\beta \in R^{C\times H\times W}$ represents the spatial coordinate weight. To generate the spatially enhanced feature map, the matrix multiplication of β and *F* is performed:

(15) $$F_{w}=\beta ⊗F$$

where $F_{w}$ is the weighted feature map with spatial information embedding. By embedding the spatial coordinate weights, SCAM effectively captures the object position information in RGB-T data under complex scenes, allowing the model to accurately filter out background pixel interference and enhance object region localization.

### 3.4. Decoder and Optimization Function

To recover the local details and global context information of RGB-T images, we construct the decoder framework based on UNet, which gradually upsamples the features and fuses the restored features from different stages. Instead of feature concatenation, we adopt feature addition as the fusion strategy to achieve a more complete integration of multi-scale information. This strategy enhances the decoder’s ability to reconstruct visual details and context features in RGB-T data. As shown in Figure 3, the decoder framework consists of four decoding units, each including a dropout layer to alleviate overfitting, two convolutional layers for transforming feature channels, and a bilinear interpolation layer for resizing feature maps. The decoding process of each unit is formally described as

(16) $$F_{i}=UP\left(\mathrm{Conv}\left(\mathrm{Conv}\left(DP\left(F_{i}\right)\right)\right)\right)+F_{i+1}$$

where UP(·) denotes the upsampling function and DP(·) represents the dropout operation. For pixel restoration, we use addition to fuse deep semantic features with shallow visual features, improving the model’s prediction accuracy.

For model optimization, we employ a weighted cross-entropy loss function [53] to supervise each segmentation class. As shown in Figure 3, CSANet performs parallel supervised training on both binary images and labeled images, resulting in a total loss function $L_{\mathrm{total}}$ composed of $L_{\mathrm{binary}}$ and $L_{\mathrm{ann}}$. Considering the singularity of binary image categories, we use weight coefficients to adjust the proportions of different loss functions. For labeled images, the pixel values are proportionally distributed in the range $\left[1,n\right]$, where *n* is the number of semantic segmentation categories. The weight $W_{i}$ reflects the proportional significance of each object category. In contrast, the pixel value range of binary images is $\left[0,1\right]$, which captures the positional information of object regions. The constructed loss functions are defined as follows:

(17) $$\begin{array}{c} J\left(\theta \right)=-\frac{1}{N}\sum_{i=1}^{N}\left[P_{i}\log{\hat{P}}_{i}+\left(1-P_{i}\right)\log\left(1-{\hat{P}}_{i}\right)\right]\\ L_{\mathrm{total}}=W_{1}\times L_{\mathrm{binary}}+W_{2}\times L_{\mathrm{ann}} \end{array}$$

where $J\left(\theta \right)=L_{\mathrm{binary}}=L_{\mathrm{ann}}$, $P_{i}\in \left\{0,1\right\}$ represents the ground truth of the *i*-th pixel, ${\hat{P}}_{i}\in \left\{0,1\right\}$ is the predicted value of the *i*-th pixel, and N=H×W is the total number of pixels in the image. To better balance the supervision tasks, we set the ratio of $W_{1}$ to $W_{2}$ as 2:1 during the optimization process. By reasonably assigning these weight coefficients, the optimal model parameters can be effectively obtained. To further clarify, the semantic label loss is used to supervise the network in distinguishing between multiple semantic categories at the pixel level, while the binary mask loss guides the network to distinguish object regions from the background by supervising a binary segmentation map. The motivation for introducing binary supervision is to enhance the network’s ability to localize object boundaries and reinforce the presence of objects, especially in challenging or ambiguous regions. This complementary supervision helps the model achieve more accurate and robust segmentation results.

## 4. Experiments and Results

### 4.1. Experimental Protocol

#### 4.1.1. Dataset

To verify the effectiveness of our method, we conducted experimental analysis on two RGB-T datasets: MFNet [44] and PST900 [46]. The MFNet dataset consists of 1569 pairs of RGB and TIR images, with 820 daytime and 749 nighttime scenes. Each image pair has a resolution of 480 × 640 pixels and includes nine object categories: car, person, bike, curve, car stop, guardrail, color cone, and bump. For the experiments, the dataset was split into three subsets, a training set (50%), a testing set (25%), and a validation set (25%), with an equal distribution of daytime and nighttime images in each subset. The PST900 dataset contains 894 pairs of RGB and TIR images with a resolution of 1280 × 720 pixels. It includes five object categories: hand drill, backpack, fire-extinguisher, survivor, and background. The dataset was divided into two subsets, a training set (66.6%) and a validation set (33.3%), ensuring an even distribution of daytime and nighttime images in both subsets. Notably, no preprocessing operations were applied to the RGB and TIR images to ensure the applicability of our CSANet in real-world scenarios.

#### 4.1.2. Evaluation Metrics

To quantitatively analyze the advantages of the proposed method, we adopt two commonly used metrics, mAcc and mIoU, to evaluate the segmentation performance of the model. The mAcc (mean Accuracy) measures the recognition accuracy of the model for each category, while the mIoU (mean Intersection over Union) comprehensively reflects the boundary positioning accuracy of the model across different object regions. The formulations are defined as follows:

(18) $$\begin{array}{c} \mathrm{mAcc}=\frac{1}{N}\sum_{i=1}^{N}\frac{TP_{i}}{TP_{i}+FN_{i}}\\ \mathrm{mIoU}=\frac{1}{N}\sum_{i=1}^{N}\frac{TP_{i}}{TP_{i}+FP_{i}+FN_{i}} \end{array}$$

where *N* denotes the number of object categories. For each category *i*, $TP_{i}$, $FP_{i}$, and $FN_{i}$ represent the true positive, false positive, and false negative counts, respectively.

#### 4.1.3. Implementation Details

The experiments were conducted using an NVIDIA A100 GPU (NVIDIA Corporation, Santa Clara, CA, USA) to implement CSANet within the PyTorch 1.2.0. framework. To improve the robustness of model training, data augmentation strategies, including random cropping and flipping, were applied to RGB and TIR image pairs. The model was optimized using the Ranger algorithm, with the initial learning rate set to $5\times 10^{-5}$. The training process spanned 500 epochs with a batch size of 4, and the momentum parameter was set to 0.9. For the feature extraction stage, the parameters were adaptively initialized using the pre-trained PVTv2-b5 model weights. During the verification and testing stages, data augmentation strategies were excluded to ensure the fairness and rationality of the experimental evaluation.

### 4.2. Comparison with State-of-the-Art Methods

#### 4.2.1. Evaluation on the MFNet Dataset

To demonstrate the effectiveness and advantages of the proposed multimodal feature extraction and fusion schemes, we compare CSANet with several state-of-the-art methods on the MFNet dataset. These methods compared include MFNet [44], RTFNet [17], PSTNet [46], MLFNet [18], FuseSeg [45], ABMDRNet [15], FEANet [19], MFFENet [54], GMNet [55], MMNet [56], EGFNet [57], MTANet [16], CCFFNet [58], CCAFFMNet [59], DSGBINet [60], CMXSegF [20], FDCNet [61], ECGFNet [62], MMSMCNet [63], LASNet [12], SFAF-MA [64], DBCNet [65], CAINet [66], U-KAN [67], and U-Mamba [68]. For CSANet, results are reported as mean ± standard deviation (STD) over five independent runs with different random seeds. For other methods, results are taken from the original papers. As shown in the results presented in Table 1 and Table 2, CSANet achieves state-of-the-art performance on the MFNet dataset, significantly outperforming existing methods in both mAcc and mIoU. Specifically, CSANet achieves an mAcc of 79.1% and an mIoU of 62.5%, surpassing the second-best MMSMCNet by 3.9% and 4.4%, respectively. These improvements highlight the effectiveness of CSANet’s multimodal feature extraction and fusion mechanism. For large-scale object categories such as Car and Person, CSANet achieves IoU scores of 85.6% and 91.0%, demonstrating its ability to accurately capture global semantic information and maintain high segmentation accuracy for dominant object regions. Moreover, CSANet excels in segmenting fine-grained and challenging objects. For instance, in the car stop category, CSANet achieves an accuracy of 64.7% and an IoU of 48.5%, which are 1.9% and 5.1% higher than the second-best results, respectively. Similarly, for the bike and guardrail categories, CSANet achieves IoU scores of 71.7% and 44.2%, outperforming other methods by substantial margins. These results demonstrate CSANet’s robustness in handling both large-scale objects and small, complex structures, ensuring accurate segmentation across diverse scene elements. Both U-KAN and U-Mamba exhibit inferior performance on the RGB-T semantic segmentation task, mainly because they lack specialized cross-modal fusion mechanisms and are not tailored to leverage the complementary characteristics of RGB and thermal data.

**Figure 7:**
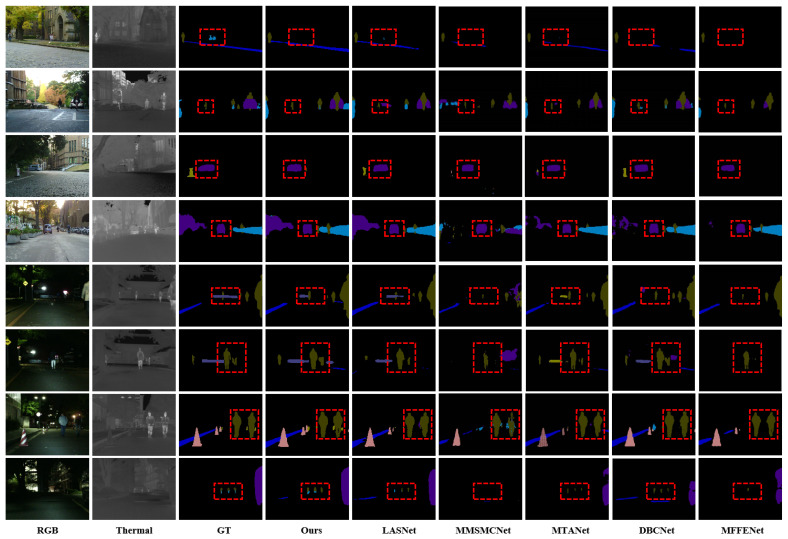
Visual comparisons of CSANet and top 5 methods in typical daytime and nighttime images on MFNet dataset.

To further evaluate CSANet’s robustness under varying illumination conditions, Table 3 compares its performance in daytime and nighttime scenes. For daytime scenes, CSANet achieves an mAcc of 76.2% and an mIoU of 61.1%, outperforming the second-best LASNet by 7.3% and 4.8%, respectively. This demonstrates CSANet’s ability to leverage multimodal data effectively in well-illuminated environments, ensuring precise segmentation of both dominant and fine-grained objects. In nighttime scenes, where RGB images suffer from significant visual degradation, CSANet achieves an mAcc of 71.9% and an mIoU of 58.3%, outperforming LASNet by 2.7% and 3.2%, respectively. These results underscore CSANet’s ability to utilize thermal (TIR) data to compensate for the deficiencies in RGB imagery, enabling robust segmentation performance under low-light conditions. Compared to other methods such as MMSMCNet and MTANet, which exhibit noticeable performance degradation in nighttime scenes, CSANet maintains high accuracy and segmentation quality, further validating its robustness in challenging real-world scenarios. For the qualitative analysis, Figure 7 provides qualitative comparisons of CSANet with the top five methods (LASNet, MMSMCNet, MTANet, DBCNet, and MFFENet) on both daytime and nighttime images. The visual results demonstrate CSANet’s superior segmentation capabilities across diverse scenarios. In daytime scenes, CSANet effectively segments both large-scale and fine-grained objects with precise boundary delineation and accurate semantic labeling. For instance, in the second row of Figure 7, CSANet accurately captures the intricate details of pedestrians and bicycles, while other methods either fail to detect these objects or produce incomplete segmentations. The advantages of CSANet are even more pronounced in nighttime scenes. In the last two rows, CSANet successfully segments challenging categories such as guardrail and bike with high precision, while competing methods struggle with incomplete or inaccurate segmentations due to the limitations of RGB imagery under low-light conditions. The integration of TIR data enables CSANet to detect and segment objects that are poorly visible in RGB images, ensuring consistent and robust performance across varying lighting conditions.

The results presented in Table 1, Table 2 and Table 3, along with the qualitative analysis in Figure 7, collectively highlight the strengths of CSANet in both quantitative and qualitative aspects. CSANet’s superior performance can be attributed to its effective multimodal feature extraction and fusion strategy, which ensures the accurate localization and segmentation of objects of varying scales, even under challenging conditions such as low illumination. Its ability to consistently outperform state-of-the-art methods in both daytime and nighttime scenes underscores its robustness and practical applicability to real-world RGB-T semantic segmentation tasks. Furthermore, the significant improvements in fine-grained segmentation tasks, such as bike, guardrail, and car stop, demonstrate CSANet’s ability to handle intricate scene elements, a critical requirement for applications such as autonomous driving and surveillance. The consistent, competitive results achieved by CSANet validate the effectiveness of its multimodal learning strategy and its ability to balance global and local feature representation, making it a promising solution for fine-grained scene understanding.

#### 4.2.2. Evaluation on PST900 Dataset

**Figure 8:**
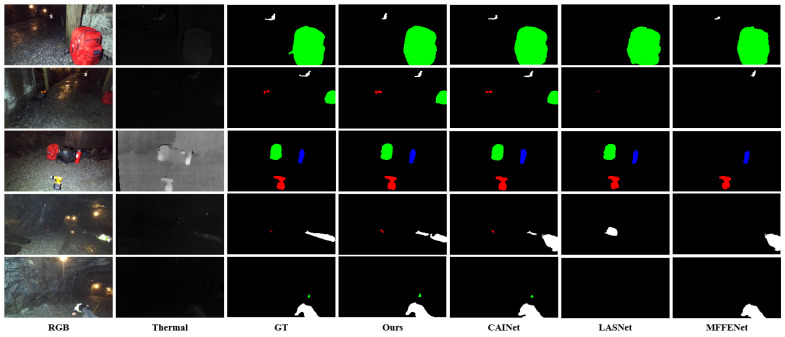
Visual comparisons of CSANet and top 3 methods on PST900 dataset.

To evaluate the generalization ability of the proposed method across diverse scenes, we conducted experiments on the PST900 dataset and compare CSANet with state-of-the-art methods, including MFNet [44], PSTNet [17], MFFENet [54], GMNet [55], EGFNet [57], MTANet [16], CCFFNet [58], DSGBINet [60], FDCNet [61], MMSMCNet [63], LASNet [12], DBCNet [65], and CAINet [66]. The quantitative results are summarized in Table 4 and Table 5, while qualitative comparisons of the top three performing methods are shown in Figure 8. As seen in Table 4 and Table 5, CSANet achieves the best overall performance, with an mAcc of 95.57% and an mIoU of 86.01%, outperforming the second-best CAINet by 1.3% in mAcc and 1.28% in mIoU. These results highlight the effectiveness of CSANet in leveraging multimodal RGB-T data to improve segmentation accuracy and robustness. CSANet demonstrates consistent superiority across most categories. Notably, for the small-scale and challenging category of fire extinguisher, CSANet achieves an IoU of 98.41%, which is 6.45% higher than the second-best CCFFNet. This significant improvement underscores the ability of CSANet to accurately segment objects with fine-grained details and complex boundaries, further enhancing overall segmentation performance. In addition to fine-grained categories, CSANet also achieves competitive results for large-scale objects such as backpacks, with an IoU of 89.88%, and for the survivor category, with an IoU of 76.51%, which is 1.02% higher than the third-best LASNet. These results further demonstrate CSANet’s robustness in capturing both global semantic contexts and detailed spatial information, making it suitable for diverse scene understanding tasks. Figure 8 presents qualitative comparisons of CSANet with CAINet, LASNet, and MFFENet. The visual results provide a more intuitive understanding of CSANet’s advantages. In the fire extinguisher category (first row), CSANet effectively delineates the object boundary and produces a more complete segmentation mask compared to other methods, which either over-segment the object or fail to capture its fine-grained details. Similarly, in the survivor category (fourth row), CSANet accurately segments the person in the scene, with minimal boundary errors, whereas CAINet and LASNet exhibit noticeable inaccuracies in the segmentation mask. Furthermore, in complex scenes with multiple objects, such as the backpack category (third row), CSANet demonstrates superior segmentation quality, correctly identifying and separating multiple objects, while other methods produce fragmented or incomplete masks. These qualitative and quantitative analyses collectively demonstrate the robustness and effectiveness of CSANet in RGB-T semantic segmentation tasks, particularly in scenarios involving small-scale objects and intricate boundaries. The ability to consistently outperform existing state-of-the-art methods highlights CSANet’s practical applicability for real-world applications, such as emergency response and autonomous systems, where accurate segmentation of diverse and challenging objects is critical.

#### 4.2.3. Computational Complexity

To evaluate the feasibility of the proposed CSANet for practical applications, we compare its computational complexity with state-of-the-art methods on the MFNet dataset.

Table 6 summarizes the floating-point operations (FLOPs) and model parameters, along with segmentation accuracy metrics (mAcc and mIoU) for different methods. CSANet achieves competitive computational efficiency with the 84.32 G FLOPs and 38.36 M parameters, which is significantly lower than methods such as RTFNet (245.71 G FLOPs, 185.24 M parameters) and PSTNet (337.04 G FLOPs, 254.51 M parameters). Notably, while CAINet has the smallest computational cost (12.16 M parameters, 67.49 G FLOPs), its segmentation performance (mAcc = 73.3%, mIoU = 58.6%) is inferior to that of CSANet (mAcc = 79.1%, mIoU = 62.5%), indicating that a lower computational cost does not necessarily result in superior segmentation accuracy. Compared to other high-performing models such as LASNet and MMSMCNet, CSANet achieves a balance between computational efficiency and segmentation accuracy. For instance, LASNet has higher FLOPs (233.81 G) and parameters (154.62 M) while achieving lower mAcc (76.8%) and mIoU (60.6%) relative to CSANet. Similarly, MMSMCNet, with 181.82 G FLOPs and 98.58 M parameters, achieves an mIoU of 58.1%, which is 4.4% lower than CSANet. These results emphasize the efficiency of CSANet in balancing model complexity and performance. While CSANet does not achieve the lowest FLOPs or parameter count among all methods, it demonstrates strong competitiveness by achieving state-of-the-art segmentation accuracy. This balance of computational cost and performance makes CSANet a practical choice for real-world applications, particularly in scenarios where hardware resources are constrained but high segmentation accuracy is required. Furthermore, the reduction in computational cost compared to other high-performing methods highlights the scalability of CSANet for deployment in tasks such as autonomous driving and surveillance. To further enhance the deployment of CSANet in resource-constrained environments such as embedded systems, mobile platforms, or real-time applications (e.g., autonomous vehicles and drones), several model optimization strategies can be considered. Techniques such as model pruning, quantization, and knowledge distillation could be employed to further reduce the computational cost and memory footprint of CSANet, enabling efficient inference on hardware with limited resources. Additionally, replacing the current backbone with a lighter variant (e.g., a smaller version of PVT or other efficient architectures) can further improve suitability for embedded deployment.

Given the current model complexity (84.32 G FLOPs, 38.36 M parameters), CSANet is expected to achieve real-time inference on high-end GPUs, with the potential for further acceleration through the aforementioned techniques. On embedded devices, with appropriate model compression and optimization, CSANet could potentially approach or meet the typical real-time requirements (e.g., 30–60 FPS) for practical applications. We consider this an important direction for future work and plan to investigate and report the detailed deployment performance of CSANet on embedded platforms.

### 4.3. Ablation Study

**Figure 9:**
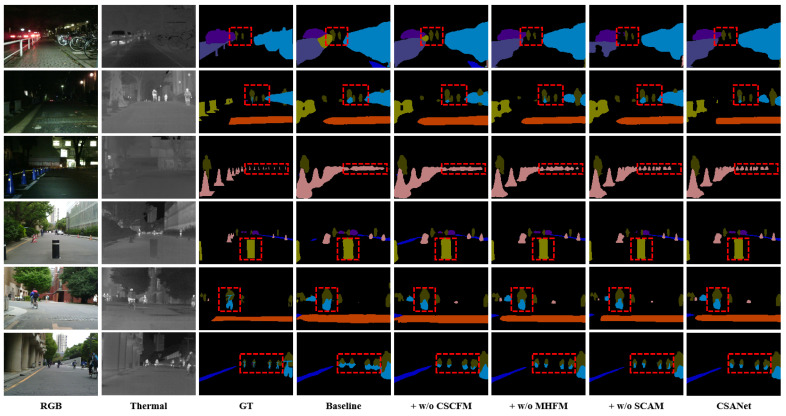
Visualized results (Baseline, +w/o CSCFM, +w/o MHFM, +w/o SCAM and CSANet) of ablation studies.

To evaluate the contributions of each component in CSANet, including the Cross-Stage Cross-Modal Feature Module (CSCFM), the Multi-Head Feature Module (MHFM), and the Spatial Context Attention Module (SCAM), we conducted ablation studies on the MFNet dataset. The results, presented in Table 7, demonstrate the significant improvements each component brings to the overall segmentation performance. The baseline model, achieving an mIoU of 53.2% and an mAcc of 64.8%, struggles to effectively handle small and complex objects, as reflected by the low IoU scores for car stops (25.6%) and poles (28.2%), as well as limited performance in categories with fine-grained boundaries, such as bikes (59.3%). Incorporating CSCFM into the baseline significantly improves the mIoU to 58.7%, with notable gains in challenging categories such as car stops (31.7%, +6.1%) and poles (34.8%, +6.6%). These results highlight CSCFM’s ability to integrate multi-scale and multi-modal features across different stages, addressing the challenges posed by scale variation and object deformation. Additionally, by adding MHFM to the baseline, the mIoU increases to 57.3%, reflecting a 4.1% improvement. This enhancement is particularly evident in categories requiring accurate boundary delineation, such as bikes (61.7%, +2.4%) and persons (89.4%, +1.2%), demonstrating MHFM’s capability to refine fine-grained boundary features. The inclusion of SCAM further boosts the mIoU to 56.8%, with consistent improvements in small-scale object categories like guardrails (35.9%, +3.2%) and car stops (29.7%, +4.1%), showcasing SCAM’s ability to extract spatial position information and enhance feature representations for small or sparsely distributed objects in complex scenes. When all three modules are fully integrated, CSANet achieves the best performance, with an mIoU of 62.5% and an mAcc of 79.1%, representing improvements of 9.3% and 14.3%, respectively, over the baseline. Across all categories, CSANet demonstrates consistent gains, particularly in small and challenging objects. For example, the IoU for car stops improves from 25.6% to 44.2%, while the IoU for poles increases from 28.2% to 39.6%. These results validate the synergistic effects of CSCFM, MHFM, and SCAM in enhancing segmentation performance for both large-scale objects (e.g., car, person) and small-scale or fine-grained objects (e.g., bike, car stop, pole), effectively balancing global semantic understanding and local detail refinement. The visualized results in Figure 9 further corroborate the quantitative findings. Compared to the baseline and ablated variants, CSANet consistently produces more accurate and complete segmentation masks, particularly for objects with complex boundaries or small scales. These results demonstrate that the combination of CSCFM, MHFM, and SCAM allows CSANet to achieve state-of-the-art performance on the MFNet dataset, addressing the challenges of multi-scale object segmentation and small-scale object recognition in RGB-T semantic segmentation tasks. To analyze the contribution of each modality, we further conducted ablation experiments using only the RGB or only the TIR modality as input. As shown in Table 8, using only RGB images, CSANet achieves an mIoU of 56.7% and mAcc of 72.4%. Using only TIR images, the model achieves an mIoU of 54.2% and mAcc of 69.8%. In contrast, the full model utilizing both modalities achieves an mIoU of 62.5% and mAcc of 79.1%. For specific categories such as cars, persons, bikes, guardrails, cars stops, and poles, the multi-modal model consistently outperforms the single-modality variants. These results clearly demonstrate that fusing RGB and thermal modalities significantly improves segmentation accuracy across all object classes, confirming the effectiveness of our multi-modal design. To further validate the effectiveness of our proposed Spatial Coordinate Attention Module (SCAM), we compare it with two widely used alternatives: Coordinate Convolution (CoordConv) and Coordinate Attention (CA). For a fair comparison, we replaced the proposed SCAM with CoordConv and CA modules in our framework and evaluated their performance on the MFNet dataset. As shown in Table 9, CSANet with CoordConv achieves an mIoU of 59.3%, and with CA, it achieves an mIoU of 60.1%, both lower than our SCAM-based model (62.5%). Moreover, our SCAM-based model consistently outperforms the others in terms of both overall (mAcc, mIoU) and per-class IoU metrics, especially for challenging categories such as guardrails, car stops, and poles. This demonstrates the superiority of SCAM in capturing spatial dependencies and enhancing segmentation quality in complex urban scenes.

## 5. Conclusions

In this study, we present the Context Spatial Awareness Network (CSANet) for RGB-T semantic segmentation tasks. CSANet introduces a novel framework that categorizes multimodal features into visual and semantic features, designing specialized extraction and fusion strategies for each feature type. Three key modules, the Cross-Stage Cross-Modal Feature Module (CSCFM), the Multi-Head Feature Module (MHFM), and the Spatial Context Attention Module (SCAM), are proposed to handle multi-scale features, refine detailed features, and enhance spatial position features, respectively. These modules enable fine-grained feature fusion while addressing the interaction challenges between feature categories and achieving efficient cross-stage feature integration. The effectiveness of CSANet is demonstrated through extensive experiments on MFNet and PST900 datasets, where it achieves state-of-the-art segmentation performance. Ablation studies further validate the contributions of each module, highlighting their complementary roles in improving segmentation accuracy and robustness. Moreover, CSANet demonstrates competitive computational complexity, balancing efficiency and performance, making it suitable for real-world applications. In future work, we plan to extend the application of CSANet to more multimodal semantic segmentation tasks, such as RGB-D or LiDAR-based segmentation, to further evaluate its robustness and generalization across diverse data modalities.

## Figures and Tables

**Table 1:** Quantitative comparisons (%) on the test set of MFNet dataset (Part 1). The top 3 results in each column are highlighted in red, green and blue. “-” denotes that the authors do not provide the corresponding results.

Methods	Years	Car		Person		Bike		Curve	
		**Acc**	**IoU**	**Acc**	**IoU**	**Acc**	**IoU**	**Acc**	**IoU**
MFNet	2017	77.2	65.9	67.0	58.9	53.9	42.9	36.2	29.9
RTFNet	2019	91.3	86.3	78.2	67.8	71.5	58.2	69.8	43.7
PSTNet	2020	-	76.8	-	52.6	-	55.3	-	29.6
MLFNet	2021	-	82.3	-	68.1	-	67.3	-	27.3
FuseSeg	2021	93.1	87.9	81.4	71.7	78.5	64.6	68.4	44.8
ABMDRNet	2021	94.3	84.8	90.0	69.6	75.7	60.3	64.0	45.1
FEANet	2021	93.3	87.8	82.7	71.1	76.7	61.1	65.5	46.5
MFFENet	2021	93.1	88.2	83.2	74.1	77.1	62.9	67.2	46.2
GMNet	2021	94.1	86.5	83.0	73.1	76.9	61.7	59.7	44.0
MMNet	2022	-	83.9	-	69.3	-	59.0	-	43.2
EGFNet	2022	95.8	87.6	89.0	69.8	80.6	58.8	71.5	42.8
MTANet	2022	95.8	88.1	90.9	71.5	80.3	60.7	75.3	40.9
CCFFNet	2022	94.5	89.6	83.6	74.2	73.2	63.1	67.2	50.5
CCAFFMNet	2022	95.2	89.1	85.9	72.5	82.3	67.5	71.8	46.3
DSGBINet	2022	95.2	87.4	89.2	69.5	85.2	64.7	66.0	46.3
CMXSegF	2022	-	89.4	-	74.8	-	64.7	-	47.3
FDCNet	2022	94.1	87.5	91.4	72.4	78.1	61.7	70.1	43.8
ECGFNet	2023	89.4	83.5	85.2	72.1	72.9	61.6	62.8	40.5
MMSMCNet	2023	96.2	89.2	93.2	69.1	83.4	63.5	74.4	46.4
LASNet	2023	94.9	84.2	81.7	67.1	82.1	56.9	70.7	41.1
SFAF-MA	2023	94.3	87.8	83.9	72.4	72.0	59.5	64.4	46.0
DBCNet	2024	93.0	87.4	82.7	73.6	70.3	61.8	71.2	47.1
CAINet	2024	93.0	88.5	74.6	66.3	85.2	68.7	65.9	55.4
U-Mamba	2024	82.7	74.3	77.9	65.5	59.9	48.8	52.4	36.2
U-KAN	2025	81.2	72.4	76.4	64.1	58.3	47.2	51.7	35.0
CSANet (Ours)	-	95.3 ± 0.2	85.6 ± 0.2	91.0 ± 0.2	65.7 ± 0.3	86.0 ± 0.2	71.7 ± 0.3	49.5 ± 0.3	37.2 ± 0.3

**Table 2:** Quantitative comparisons (%) on the test set of MFNet dataset (Part 2). The top 3 results in each column are highlighted in red, green and blue. “-” denotes that the authors do not provide the corresponding results.

Methods	Years	Car Stop		Guardrail		Color Cone		Bump		mAcc	mIoU
		**Acc**	**IoU**	**Acc**	**IoU**	**Acc**	**IoU**	**Acc**	**IoU**		
MFNet	2017	19.1	9.9	0.1	8.5	30.3	25.2	30.0	27.7	45.1	39.7
RTFNet	2019	32.1	24.3	13.4	3.6	40.4	26.0	73.5	57.2	62.2	51.7
PSTNet	2020	-	25.1	-	15.1	-	39.4	-	45.0	-	48.4
MLFNet	2021	-	30.4	-	15.7	-	55.6	-	40.1	-	53.8
FuseSeg	2021	29.1	22.7	63.7	6.4	55.8	46.9	66.4	47.9	70.6	54.5
ABMDRNet	2021	44.1	33.1	31.0	5.1	61.7	47.4	66.2	50.0	69.5	54.8
FEANet	2021	26.6	22.1	70.8	6.6	66.6	55.3	77.3	48.9	73.2	55.3
MFFENet	2021	52.3	37.1	65.0	7.6	58.5	52.4	73.4	47.4	74.3	57.1
GMNet	2021	55.0	42.3	71.2	14.5	54.7	48.7	73.1	47.4	74.1	57.3
MMNet	2022	-	24.7	-	4.6	-	42.2	-	50.7	62.7	52.8
EGFNet	2022	48.7	33.8	33.6	7.0	65.3	48.3	71.1	47.1	72.7	54.8
MTANet	2022	62.8	38.9	38.7	13.7	63.8	45.9	70.8	47.2	75.2	56.1
CCFFNet	2022	38.7	31.9	30.6	4.8	55.2	49.7	72.9	56.3	68.3	57.6
CCAFFMNet	2022	32.5	25.2	56.8	17.3	58.3	50.6	76.6	58.3	72.9	58.2
DSGBINet	2022	56.7	43.4	7.8	3.3	82.0	61.7	72.8	48.9	72.6	58.1
CMXSegF	2022	-	30.1	-	8.1	-	52.4	-	59.4	-	58.2
FDCNet	2022	34.4	27.2	61.5	7.3	64.0	52.0	74.5	56.6	74.1	56.3
ECGFNet	2023	44.8	30.8	45.2	11.1	57.2	49.7	65.1	50.9	69.1	55.3
MMSMCNet	2023	56.6	41.9	26.9	8.8	70.2	48.8	77.5	57.6	75.2	58.1
LASNet	2023	56.8	39.6	59.5	18.9	58.1	48.8	77.2	40.1	75.4	54.9
SFAF-MA	2023	34.0	24.7	35.6	4.3	55.8	39.1	67.9	52.6	67.5	53.8
DBCNet	2024	46.2	33.8	78.2	62.9	57.7	50.9	74.6	45.4	74.8	56.2
CAINet	2024	34.7	31.5	65.6	9.0	55.6	48.9	85.0	60.7	73.2	58.6
U-Mamba	2024	29.5	18.1	41.6	24.3	62.7	42.8	48.8	38.3	63.5	48.2
U-KAN	2025	28.2	16.6	40.1	22.7	61.4	41.2	47.6	37.0	62.1	46.7
CSANet (Ours)	-	64.7 ± 0.4	48.5 ± 0.3	78.2 ± 0.3	44.2 ± 0.3	87.0 ± 0.2	58.9 ± 0.3	62.1 ± 0.2	53.5 ± 0.2	79.1 ± 0.15	62.5 ± 0.13

**Table 3:** Quantitative comparison (%) of daytime and nighttime scenes.

Methods	Daytime		Nighttime	
	**mAcc**	**mIoU**	**mAcc**	**mIoU**
DSGBINet	62.8	47.2	65.3	48.9
FEANet	64.7	52.3	61.5	44.2
CAINet	66.4	53.9	60.2	41.3
GMNet	65.1	52.7	63.4	46.5
FDCNet	63.6	49.2	61.7	44.9
MFFENet	66.5	52.3	64.9	47.6
DBCNet	67.2	54.5	62.3	45.1
MTANet	65.8	53.4	61.2	43.9
MMSMCNet	66.9	54.7	62.0	44.6
LASNet	68.9	56.3	69.2	55.1
CSANet (Ours)	76.2	61.1	71.9	58.3

**Table 4:** Quantitative comparisons (%) on the test set of the PST900 dataset (Part 1). The top 3 results in each column are highlighted in red, green and blue. “-” denotes that the authors do not provide the corresponding results.

Methods	Years	Background		Hand-Drill		Backpack	
		**Acc**	**IoU**	**Acc**	**IoU**	**Acc**	**IoU**
MFNet	2017	-	98.63	-	41.13	-	64.27
PSTNet	2020	-	98.85	-	53.60	-	69.20
MFFENet	2021	-	99.40	-	72.50	-	81.02
GMNet	2021	99.81	99.44	90.29	85.17	89.01	83.82
EGFNet	2022	99.48	99.26	97.99	64.67	94.17	83.05
MTANet	2022	-	99.33	-	62.05	-	87.50
CCFFNet	2022	99.9	99.4	89.7	82.8	77.5	75.8
DSGBINet	2022	99.73	99.39	94.53	74.99	88.65	85.11
FDCNet	2022	99.72	99.15	82.52	70.36	77.45	72.17
MMSMCNet	2023	99.55	99.39	97.96	62.36	96.94	89.22
LASNet	2023	99.77	99.46	91.81	82.80	90.80	86.48
DBCNet	2024	-	99.40	-	77.19	-	82.67
CAINet	2024	99.66	99.50	95.87	80.30	96.09	88.02
CSANet (Ours)	-	99.73	99.55	95.65	83.76	97.90	89.88

**Table 5:** Quantitative comparisons (%) on the test set of PST900 dataset (Part 2). The top 3 results in each column are highlighted in red, green and blue. “-” denotes that the authors do not provide the corresponding results.

Methods	Years	Fire-Extinguisher		Survivor		mAcc	mIoU
		**Acc**	**IoU**	**Acc**	**IoU**		
MFNet	2017	-	60.35	-	20.70	-	57.02
PSTNet	2020	-	70.12	-	50.03	-	68.36
MFFENet	2021	-	66.38	-	75.60	-	78.98
GMNet	2021	88.28	73.79	80.86	78.36	89.61	84.12
EGFNet	2022	95.17	71.29	83.30	74.30	94.02	78.51
MTANet	2022	-	64.95	-	79.14	-	78.60
CCFFNet	2022	87.6	79.9	79.7	72.7	86.9	82.1
DSGBINet	2022	94.78	79.31	81.37	75.56	91.81	82.87
FDCNet	2022	91.77	71.52	78.36	72.36	85.96	77.11
MMSMCNet	2023	97.36	73.29	84.28	74.70	95.20	79.80
LASNet	2023	92.36	77.75	83.43	75.49	91.63	84.40
DBCNet	2024	-	72.95	-	76.68	-	81.78
CAINet	2024	88.38	77.21	91.35	78.69	94.27	84.73
CSANet (Ours)	-	98.41	86.35	86.18	76.51	95.57	86.01

**Table 6:** Computational complexity comparison of different methods.

Methods	Input Size	FLOPs/G ↓	Params/M ↓	mAcc	mIoU
RTFNet	640 × 480	245.71	185.24	62.2	51.7
PSTNet	640 × 480	337.04	254.51	-	48.4
FuseSeg	640 × 480	129.37	20.38	70.6	54.6
ABMDRNet	640 × 480	194.33	64.60	69.5	54.8
EGFNet	640 × 480	201.29	62.82	72.7	54.8
MTANet	640 × 480	264.69	121.58	75.2	56.1
FDCNet	640 × 480	159.05	52.91	74.1	56.3
MMSMCNet	640 × 480	181.82	98.58	75.2	58.1
LASNet	640 × 480	233.81	93.58	75.4	54.9
DBCNet	640 × 480	67.49	47.87	74.8	56.2
CAINet	640 × 480	123.62	12.16	73.2	58.6
CSANet (Ours)	640 × 480	84.32	38.36	79.1	62.5

**Table 7:** Quantitative results (%) of ablation studies on the MFNet dataset. The results include overall metrics (mAcc and mIoU) and per-class IoU.

Variants	mAcc	mIoU	Car	Person	Bike	Guardrail	Car Stop	Pole
Baseline	64.8	53.2	85.1	88.2	59.3	32.7	25.6	28.2
Baseline + w/o CSCFM	76.9	58.7	86.8	90.1	62.5	36.4	31.7	34.8
Baseline + w/o MHFM	69.5	57.3	86.0	89.4	61.7	35.8	30.2	33.5
Baseline + w/o SCAM	75.3	56.8	86.4	89.8	62.1	35.9	29.7	31.2
CSANet (Ours)	79.1	62.5	87.6	91.0	65.7	37.2	44.2	39.6

**Table 8:** Quantitative results (%) of single-modality and multi-modality ablation studies on the MFNet dataset.

Variants	mAcc	mIoU	Car	Person	Bike	Guardrail	Car Stop	Pole
CSANet (RGB only)	72.4	56.7	82.1	85.9	61.2	33.5	35.8	32.1
CSANet (TIR only)	69.8	54.2	80.4	83.6	59.4	31.2	33.6	30.8
CSANet (RGB+TIR)	79.1	62.5	87.6	91.0	65.7	37.2	44.2	39.6

**Table 9:** Quantitative results (%) of different attention modules on the MFNet dataset.

Variants	mAcc	mIoU	Car	Person	Bike	Guardrail	Car Stop	Pole
CSANet w/CoordConv	76.8	59.3	81.2	88.3	62.1	38.5	38.7	35.8
CSANet w/CoordAtt	77.5	60.1	82.3	89.1	63.0	39.6	39.8	36.5
CSANet w/SCAM	79.1	62.5	87.6	91.0	65.7	37.2	44.2	39.6

## Data Availability

The data presented in this study are openly available in https://github.com/darkseid-arch/CSANet (accessed on 8 April 2025).
